# Secondary Pseudohypoaldosteronism Associated With Mild Hydronephrosis in a Newborn

**DOI:** 10.7759/cureus.13462

**Published:** 2021-02-20

**Authors:** Kazuki Kodo, Sachiko Goto, Yoshiki Katsumi

**Affiliations:** 1 Department of Pediatrics, Saiseikai Kyoto Hospital, Kyoto, JPN

**Keywords:** secondary pseudohypoaldosteronism, mild hydronephrosis, hyperkalemia, hyponatremia

## Abstract

Neonatal hyponatremia with hyperkalemia is a rare but potentially life-threatening occurrence. Aldosterone deficiency secondary to congenital adrenal hyperplasia (CAH) is often suspected in these cases, although it is not easy to accurately diagnose it initially. We report the case of a 12-day-old female infant presenting with poor sucking, hyperkalemia, and hyponatremia. Plasma renin activity (PRA) and aldosterone levels were markedly elevated, and mild hydronephrosis [Society for Fetal Urology (SFU) grade 1] was noted. We then suspected secondary pseudohypoaldosteronism (S-PHA); however, her serum potassium level remained elevated despite sodium infusion. Because we could not rule out a diagnosis of adrenal insufficiency caused by CAH, we cautiously initiated hydrocortisone. After reviewing the results of a mass screening test and a urine steroid profile analysis, adrenal diseases were ruled out and we diagnosed the patient with S-PHA. This report aims to illustrate that mild hydronephrosis can cause S-PHA by inducing renal tubular resistance to aldosterone. Because the symptoms of S-PHA are similar to those of CAH, we recognize that further studies are needed to clarify their differences.

## Introduction

Pseudohypoaldosteronism (PHA) is classified into primary and secondary PHA (S-PHA). Primary PHA is generally characterized by mutations in the mineralocorticoid receptor, and S-PHA is associated with urinary tract abnormalities and/or urinary tract infection (UTI), leading to renal or generalized resistance to aldosterone [1]. However, the mechanism of aldosterone resistance is not completely understood [1-3]. These conditions induce hyponatremia with hyperkalemia and can occasionally lead to a life-threatening condition in infants. Adrenal diseases such as congenital adrenal hyperplasia (CAH) should be considered when making a differential diagnosis [4-5]. In this report, we discuss the case of a 12-day-old female infant presenting with poor sucking, hyperkalemia, and hyponatremia who was diagnosed with S-PHA associated with mild hydronephrosis, which was difficult to distinguish from CAH in the early stages.

## Case presentation

**Figure 1:**
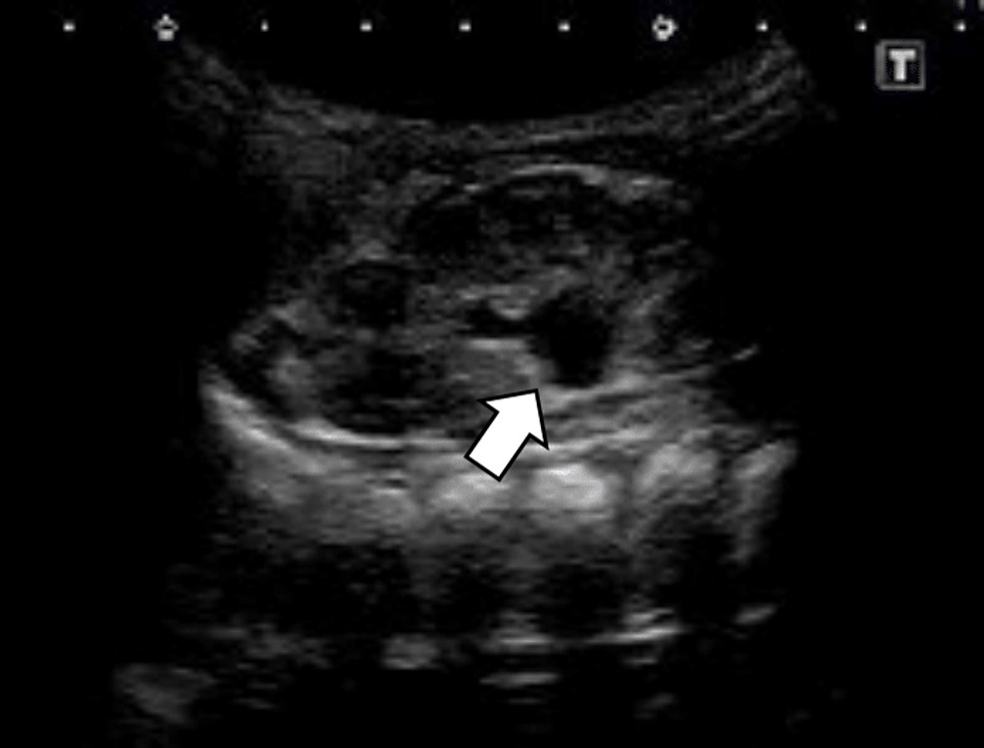
Ultrasound imaging Ultrasonography showing a mild expansion of the renal pelvis of the left kidney (arrow), which was classified as mild hydronephrosis based on the Society for Fetal Urology (SFU) grading system (grade 1)

A 12-day-old female infant presented with a history of poor sucking and was hospitalized for further examination of hyperkalemia and hyponatremia. She had been born at full term with a birth weight of 3,326 g and had shown no symptoms at birth. When she was brought to our hospital, her weight was 3,339 g. As the skin turgor and capillary refill time were normal, no obvious dehydration was observed. Neither hyperpigmentation of the skin nor virilization of the external genitalia was observed. Laboratory test results were as follows: serum sodium: 134 mEq/L; serum potassium: 6.2 mEq/L; serum glucose: 72 mg/dL; blood urea nitrogen (BUN): 12 mg/dL; and creatinine: 0.49 mg/dL. Metabolic acidosis was not noted. Urinalysis results were normal. Urinary sodium and potassium levels were 12 mmol/L [fractional excretion of sodium (FeNa) was 0.68%] and 16 mmol/L [fractional excretion of potassium (FeK) was 1.98%], respectively (Table 1). Abdominal ultrasonography revealed mild hydronephrosis [Society for Fetal Urology (SFU) grade 1] in the left kidney (Figure 1). The infant’s feeding was dramatically improved by administering intravenous saline. The serum sodium level increased to 137 mEq/L; however, the serum potassium level was still above 6.0 mEq/L. Hormone evaluation results were as follows: adrenocorticotropic hormone (ACTH): 50.3 pg/mL; cortisol: 25.4 μg/dL; plasma renin activity (PRA): 36.7 ng/mL/h; and aldosterone: 1,678.2 pg/mL (Table 1). PRA and aldosterone levels were markedly elevated, suggesting profound tubular resistance to aldosterone. Hence, we suspected the presence of S-PHA with hydronephrosis.

**Table 1:** Laboratory findings WBC: white blood cell; Hb: hemoglobin; Hct: hematocrit; Plt: platelets; BE: base excess; LDH: lactate dehydrogenase; Alb: albumin; BUN: blood urea nitrogen; Cre: creatinine; Na: sodium; K: potassium; Cl: chloride; Ca: calcium; T-bil: total bilirubin; AST: aspartate aminotransferase; ALT: alanine aminotransferase; Glu: glucose; PRA: plasma renin activity; ACTH: adrenocorticotropic hormone; 17-OHP: 17-hydroxyprogesterone; FeNa: fractional excretion of sodium; FeK: fractional excretion of potassium

Test	Value		Normal range		Test	Value			Normal range
Hematology					Venous blood gas				
WBC	12,200	/μL	(3,500-9,100)		pH		7.439	mmHg	(7.36-7.44)
Hb	17.3	g/dL	(11.3-15.2)		pCO_2_		32.1	mmol/L	(36-44)
Hct	48.2	%	(33.4-44.9)		HCO_3_		22.7	mmol/L	(22-26)
Plt	62.4	×10^4^/μL	(13.0-36.9)		BE		-1.6		
Biochemistry					Endocrine				
LDH	304	IU/L	(115-245)		Cortisol		25.4	μg/dl	(4.5-21.1)
Alb	4	g/dL	(3.8-5.3)		Aldosterone		1,678.20	pg/mL	(35.7-240)
BUN	12	mg/dL	(8-22)		PRA		36.7	ng/mL/h	(0.2-2.7)
Cre	0.49	mg/dL	(0.47-0.79)		ACTH		50.3	pg/mL	(7.4-55.7)
Na	134	mEq/L	(135-147)		17-OHP		2	ng/ml	(0.6-2.8)
K	6.2	mEq/L	(3.6-5.0)		Urine				
Cl	104	mEq/L	(98-108)		Na		12	mEq/L	(15-260)
Ca	11.5	mg/dL	(8.6-10.1)		K		16	mEq/L	(3.8-160)
T-bil	8.6	mg/dL	(0.2-1.1)		Cre		6.4	mEq/L	(0.6-400)
AST	24	IU/L	(10-40)		FeNa		0.68	%	
ALT	16	IU/L	(5-45)		FeK		1.98	%	
Glu	72	mg/dL	(50-109)						

**Figure 2:**
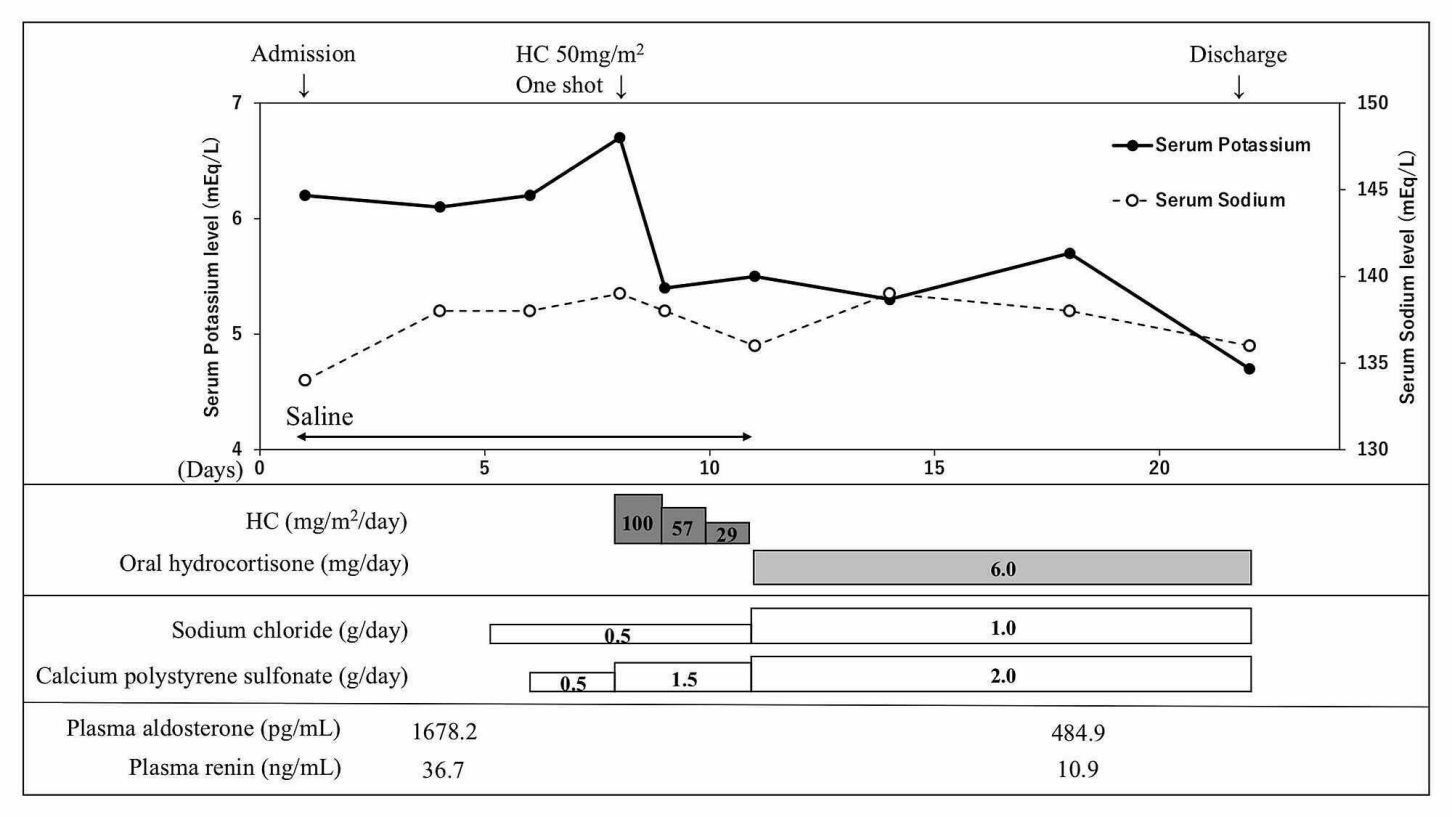
The patient’s course of hospitalization HC: hydrocortisone

On day four of her admission (16 days since birth), oral sodium chloride supplementation, as well as calcium polystyrene sulfonate, was started. However, the serum potassium level increased to 6.7 mEq/L, and an electrocardiogram revealed the peaking of T waves. Hydrocortisone (50 mg/m^2^) was intravenously administered, and the serum potassium level rapidly decreased to 5.4 mEq/L on the following day. With a presumptive diagnosis of CAH, we continued treatment with hydrocortisone. The infant’s electrolytes remained normal on follow-up. However, a mass screening test performed on day four showed a very low concentration of 17-hydroxyprogesterone (17-OHP; direct method) of 2.0 ng/mL. To exclude a diagnosis of adrenal insufficiency, we performed a urine steroid profile analysis for further consideration. During that time, we continued steroid administration along with oral hydrocortisone. The patient was temporarily discharged on day 22 of admission (34 days since birth) and was prescribed oral hydrocortisone of 6 mg/day, sodium chloride supplementation of 1.0 g/day, and calcium polystyrene sulfonate of 2.0 g/day (Figure 2). We confirmed that the urine steroid profile was normal, and steroid administration was gradually decreased. At six months of age, we discontinued all drugs after confirming that there had been no recurrence of electrolyte abnormalities and that there were no symptoms induced by high renin or high aldosterone levels. The patient is currently one year old. She has been followed up with no problems noted other than the continued but mild hydronephrosis.

## Discussion

S-PHA is strongly associated with urinary tract abnormalities, differentiating it from primary PHA, which is associated with genetics. S-PHA can exhibit transient renal tubular resistance to aldosterone; however, the mechanism of aldosterone resistance remains unclear [1-3]. The risk of S-PHA diminishes substantially after three months of age. A case review of infants with S-PHA showed that 80% had UTI with urinary tract abnormalities, 12% had only urinary tract abnormalities, and the remaining 8% had isolated incidences of UTI. These findings highlight the fact that in most cases, pyelonephritis alone cannot cause a clinically relevant sodium loss unless it occurs in conjunction with a congenital urinary tract malformation of any degree [5]. Nishida et al. have reported that even mild hydronephrosis can cause S-PHA, which can be exacerbated by UTI [6].

PHA may present with nonspecific symptoms, such as poor weight gain, poor feeding, or dehydration, but sometimes serious symptoms that are associated with high potassium levels, such as shock and fatal arrhythmia, can occur [7]. In our case, we detected hyponatremia and hyperkalemia, together with poor weight gain and poor feeding. Further examination found high renin and aldosterone levels with mild hydronephrosis, which led to the diagnosis of S-PHA. Despite early intervention with sodium administration, the infant’s potassium level gradually increased, and we suspected CAH. In such cases, it is extremely important to consider that CAH can present with hyponatremia and hyperkalemia. There are specific symptoms characteristic of CAH, such as ambiguous genitalia in girls and hyperpigmentation of the skin, but there are no specific symptoms associated with PHA. For CAH, corticosteroids need to be administered, whereas, for PHA, corticosteroids need not be administered. Eventually, we administered hydrocortisone in our case, despite the early diagnosis of PHA.

Some PHA patients are misdiagnosed with CAH and are treated with corticosteroids [3,5]. Initially, it is very difficult to distinguish PHA from CAH in a clinical setting. It is important to evaluate the pathophysiology of aldosterone resistance to distinguish between PHA and CAH. Serum aldosterone and cortisol levels are expected to be low in patients with CAH, while elevated 17-OHP and ACTH levels are important markers for diagnosis. In contrast, in patients with PHA, serum aldosterone levels are significantly elevated, while 17-OHP and serum cortisol levels are expected to be normal. However, previous reports have indicated that CAH patients sometimes have normal to very high aldosterone levels and normal cortisol levels, which can make the diagnosis and initial treatment of CAH difficult [8]. Thus, a diagnosis cannot be reached based on blood test results alone. Measurement of urinary sodium levels may reveal excessive urinary sodium excretion. Measurement of the urinary potassium level, FeK, and transtubular potassium gradient (TTKG) may reveal the pathophysiology of aldosterone resistance [9]. Therefore, it is necessary to diagnose patients comprehensively. If there are urinary tract abnormalities, you may simply presume the existence of aldosterone resistance. Ultimately, an ACTH stimulation test is required to distinguish PHA from CAH, although it is very difficult to obtain frequent blood samples from neonates. Genetic analysis is an alternative diagnostic method, but it takes too long to get the results [10].

Regarding past reports of S-PHA, many cases have required either antibiotic treatment for UTI or surgical correction of a urinary tract deformity [3]. However, in our case, both the symptoms and laboratory test abnormalities were mild, and there was no evidence of UTI in the urinalysis. The hydronephrosis in our case was milder than that in previous cases. Nishida et al. have claimed that PHA can occur with only slight hydronephrosis and without UTI [6]. In our case, PHA developed without being accompanied only by such a minimal renal lesion. If UTI had accompanied these conditions, the patient's state would have worsened. UTI appears to be an exacerbating factor for aldosterone resistance. Furthermore, we contemplated the effectiveness of the steroid treatment. The patient’s condition was poor due to poor sucking. We assumed that the secretion of mineralocorticoids was relatively lacking.

The existence of mild hydronephrosis in newborn babies is a common symptom. We suggest that even mild hydronephrosis can cause the pathophysiology of aldosterone resistance, resulting in S-PHA.

## Conclusions

The presence of hyponatremia and hyperkalemia with mild hydronephrosis in the patient enabled us to diagnose S-PHA at an early stage, although it was difficult to exclude CAH. Even if hydronephrosis is mild, S-PHA should be considered.

## References

[1] Tajima T, Morikawa S, Nakamura A (2017). Clinical features and molecular basis of pseudohypoaldosteronism type 1. Clin Pediatr Endocrinol.

[2] Krishnappa V, Ross JH, Kenagy DN, Raina R (2016). Secondary or transient pseudohypoaldosteronism associated with urinary tract anomaly and urinary infection: a case report. Urol Case Rep.

[3] Bogdanović R, Stajić N, Putnik J, Paripović A (2009). Transient type 1 pseudo-hypoaldosteronism: report on an eight-patient series and literature review. Pediatr Nephrol.

[4] Ishii T, Anzo M, Adachi M (2015). Guidelines for diagnosis and treatment of 21-hydroxylase deficiency (2014 revision). Clin Pediatr Endocrinol.

[5] Sethi SK, Wazir S, Bansal S, Khokhar S, Wadhwani N, Raina R (2018). Secondary pseudohypoaldosteronism masquerading congenital adrenal hyperplasia in a neonate. Kidney Int Rep.

[6] Nishida K, Fujioka K, Ashina M (2019). A newborn case of secondary pseudohypoaldosteronism caused by a mild unilateral hydronephrosis (link in Japanese). Jpn J Pediatr.

[7] Rajpoot SK, Maggi C, Bhangoo A (2014). Pseudohypoaldosteronism in a neonate presenting as life-threatening arrhythmia. Endocrinol Diabetes Metab Case Rep.

[8] Balcells C, Gili T, Pérez J, Corripio R (2013). Pseudohypoaldosteronism without nephropathy masking salt-wasting congenital adrenal hyperplasia genetically confirmed. BMJ Case Rep.

[9] Choi MJ, Ziyadeh FN (2008). The utility of the transtubular potassium gradient in the evaluation of hyperkalemia. J Am Soc Nephrol.

[10] Ağladıoğlu SY, Aycan Z, Kendirci HN, Erkek N, Baş VN (2011). Does pseudohypoaldosteronism mask the diagnosis of congenital adrenal hyperplasia?. J Clin Res Pediatr Endocrinol.

